# protti: an R package for comprehensive data analysis of peptide- and protein-centric bottom-up proteomics data

**DOI:** 10.1093/bioadv/vbab041

**Published:** 2021-12-10

**Authors:** Jan-Philipp Quast, Dina Schuster, Paola Picotti

**Affiliations:** 1 Department of Biology, Institute of Molecular Systems Biology, ETH Zurich, Zurich 8093, Switzerland; 2 Department of Biology, Institute of Molecular Biology and Biophysics, ETH Zurich, Zurich 8093, Switzerland; 3 Laboratory of Biomolecular Research, Division of Biology and Chemistry, Paul Scherrer Institute, Villigen 5232, Switzerland

## Abstract

**Summary:**

We present a flexible, user-friendly R package called *protti* for comprehensive quality control, analysis and interpretation of quantitative bottom-up proteomics data. protti supports the analysis of protein-centric data such as those associated with protein expression analyses, as well as peptide-centric data such as those resulting from limited proteolysis-coupled mass spectrometry analysis. Due to its flexible design, it supports analysis of label-free, data-dependent, data-independent and targeted proteomics datasets. protti can be run on the output of any search engine and software package commonly used for bottom-up proteomics experiments such as Spectronaut, Skyline, MaxQuant or Proteome Discoverer, adequately exported to table format.

**Availability and implementation:**

protti is implemented as an open-source R package. Release versions are available via CRAN (https://CRAN.R-project.org/package=protti) and work on all major operating systems. The development version is maintained on GitHub (https://github.com/jpquast/protti). Full documentation including examples is provided in the form of vignettes on our package website (jpquast.github.io/protti/).

## 1 Introduction

With novel and evolving proteomics methods such as limited proteolysis coupled to mass spectrometry (LiP-MS; Feng *et al.*, 2014), N-terminomics, phosphoproteomics and other post-translational modification-centric mass spectrometry-based experiments, all of which require peptide-centric data analysis, there is a growing need for flexible software tools to analyse bottom-up proteomics data. Data structures of such bottom-up proteomics experiments are diverse, and users often require specific workflows. Existing bottom-up proteomics R packages such as MSstats (Choi *et al.*, 2014), DEP (Zhang *et al.*, 2018), msmsEDA (Gregori *et al.*, 2021), MSnbase (Gatto *et al.*, 2021), TPP (Childs *et al.*, 2021), HaDeX (Puchala *et al.*, 2020) and NormalyzerDE (Willforss *et al.*, 2019) either offer fixed analysis pipelines (e.g. packages of the MSstats family) and are therefore not easily implementable for specific user needs or are not suited for comprehensive peptide-level analysis, especially of LiP-MS data, since they offer a limited set of functions (DEP, msmsEDA, MSnbase, TPP, HaDeX, NormalyzerDE). Finally, few available tools offer functions for quality control, data analysis and data interpretation in one package.

To fill this gap, we developed protti, a user-friendly label-free bottom-up proteomics R package that facilitates the analysis of data-dependent, data-independent and targeted mass spectrometry data on the protein, peptide, precursor or fragment level. protti provides flexible functions for quality control, data filtering, differential quantification and dose-response analysis that can be tailored to a user’s needs, as well as supporting biological interpretation such as functional enrichment and network analysis. protti follows the design principles of the tidyverse package family (Wickham *et al.*, 2019), which makes it accessible and easy to modify even for inexperienced R users; the code is written with the novice user in mind, and is carefully documented. Due to its modular design, most functions can be used independently of each other and can be applied to input data from many sources. Although protti does not provide a graphical user interface, unlike proteomics tools such as proteosign (Efstathiou *et al.*, 2017), Perseus (Tyanova and Cox, 2018), Prostar (Wieczorek *et al.*, 2017), DAnTE (Karpievitch *et al.*, 2009), PIQMIe (Kuzniar and Kanaar, 2014), StatQuant (van Breukelen *et al.*, 2009), LFQ-Analyst (Shah *et al.*, 2020), ProtExA (Minadakis *et al.*, 2020) and MSqRob (Goeminne *et al.*, 2018) this aids the seamless implementation of protti into any R data analysis workflow.

## 2 Overview

**Fig. 1.**
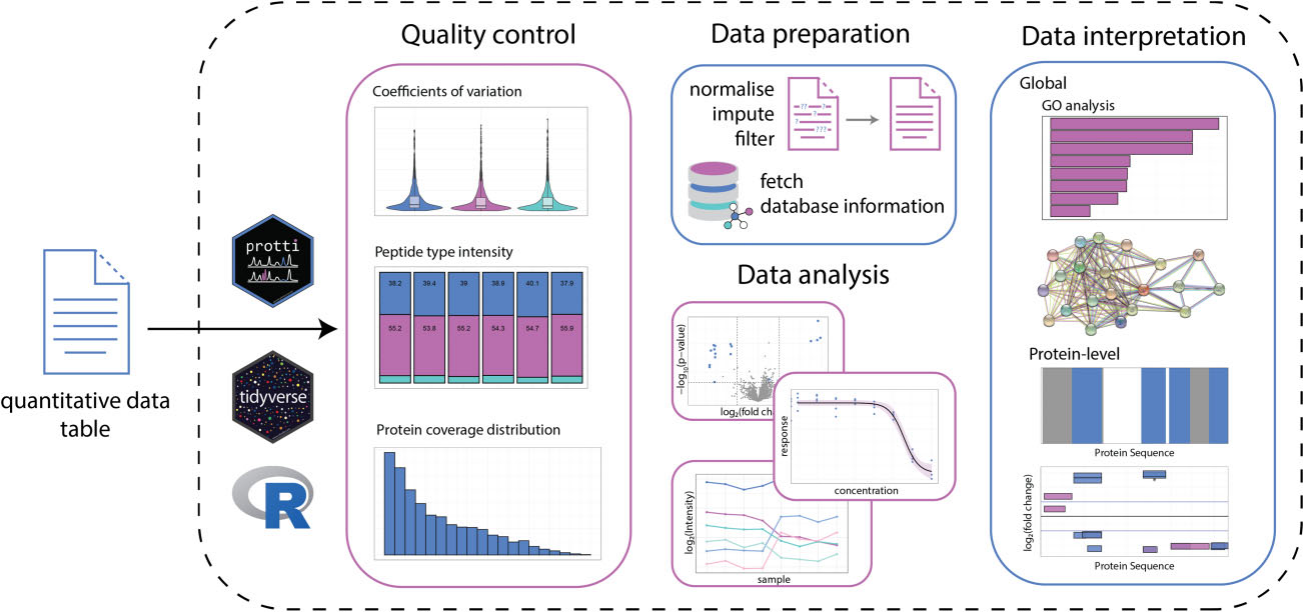
Overview of protti functions. protti can be used on the output of any software package for quantitative analysis of bottom-up proteomics experiments and provides a flexible set of functions for quality control, as well as data pre-processing, data analysis and data interpretation

protti provides a wide range of functions (Fig. 1). Briefly, the user can assess and visualize data quality and perform median normalization to correct for unequal sample amounts. Protein abundances can be inferred from precursor or peptide intensities based on methods adapted from the MaxLFQ algorithm (Cox *et al.*, 2014) and as previously implemented in the R package iq (Pham *et al.*, 2020). Differential abundance calculation in conjunction with statistical testing can be applied to any case-control dataset. Statistical testing can either be performed using a standard Welch's t-test, ANOVA, a moderated t-test based on the limma R package (Ritchie *et al.*, 2015) or the proDA R package (Ahlmann-Eltze, 2020). Dose-response experiments can be analysed by fitting four-parameter log-logistic regression models to the data based on functions from the drc R package (Ritz *et al.*, 2015).

Lists of significantly altered proteins obtained with any of the described workflows, can be further analysed using functional (Ashburner *et al.*, 2000) or pathway enrichment analyses, as well as a network analysis tool based on the STRINGdb R package (Szklarczyk *et al.*, 2019). Furthermore, to obtain additional information on proteins, including PTMs, subcellular location, PDB structures, and interaction partners, data can be annotated using information imported from several databases (UniProt (The UniProt Consortium, 2021), KEGG (Kanehisa and Goto, 2000), MobiDB (Piovesan *et al.*, 2021), ChEBI (Hastings *et al.*, 2016)). With the respective fetch functions, the user can load this information directly into R. Functions for the analysis of peptide-centric data from LiP-MS experiments allow visualiztion of differentially abundant peptides along the protein sequence.

### 2.1 Applications

protti can be applied to any bottom-up label-free proteomics dataset. In principle, the output of any table-formatted proteomic quantitative data analysis software is supported if it contains information on sample, condition, intensity, protein ID (UniProt ID), and the feature the measured intensity reports on (fragment, precursor, peptide) if different from protein intensity. The quality control, data analysis and data interpretation modules of protti can be used serially or independently.

### 2.2 Implementation

The modularity of protti enables its use in conjunction with user-defined workflows and functions. protti is written as simply as possible using tidyverse packages (Wickham *et al.*, 2019), making its source code easy to understand and adapt or extend, even for inexperienced R users.

## 3 Conclusions

We describe protti, a flexible, user-friendly R package for analysis of various bottom-up proteomics datasets. protti provides functions for quality control, data analysis on protein, peptide, precursor or fragment levels, as well as data interpretation within a single, easy-to-use package. We include examples of data analysis workflows as vignettes to illustrate the utility of the different functions for the analysis of protein- or peptide-centric datasets.

## References

[vbab041-B1] Ahlmann-Eltze C. (2020) *Differential Abundance Analysis of Label-Free Mass Spectrometry Data*. R package version 1.4.0.

[vbab041-B2] Ashburner M. et al (2000) Gene ontology: tool for the unification of biology. The Gene Ontology Consortium. Nat. Genet., 25, 25–29. 10802651 10.1038/75556 PMC3037419

[vbab041-B3] Childs D. et al (2021) TPP: Analyze Thermal Proteome Profiling (TPP) Experiments. R package version 3.20.1.

[vbab041-B4] Choi M. et al (2014) MSstats: an R package for statistical analysis of quantitative mass spectrometry-based proteomic experiments. Bioinformatics, 30, 2524–2526. 24794931 10.1093/bioinformatics/btu305

[vbab041-B5] Cox J. et al (2014) Accurate proteome-wide label-free quantification by delayed normalization and maximal peptide ratio extraction, termed MaxLFQ. Mol. Cell Proteomics, 13, 2513–2526. 24942700 10.1074/mcp.M113.031591 PMC4159666

[vbab041-B6] Efstathiou G. et al (2017) ProteoSign: an end-user online differential proteomics statistical analysis platform. Nucleic Acids Res., 45, W300–W306. 28520987 10.1093/nar/gkx444 PMC5793730

[vbab041-B7] Feng Y. et al (2014) Global analysis of protein structural changes in complex proteomes. Nat. Biotechnol., 32, 1036–1044. 25218519 10.1038/nbt.2999

[vbab041-B8] Gatto L. et al (2021) MSnbase, efficient and elegant R-based processing and visualization of raw mass spectrometry data. J. Proteome Res., 20, 1063–1069. 32902283 10.1021/acs.jproteome.0c00313

[vbab041-B9] Goeminne L.J.E. et al (2018) Experimental design and data-analysis in label-free quantitative LC/MS proteomics: a tutorial with MSqRob. J. Proteomics, 171, 23–36. 28391044 10.1016/j.jprot.2017.04.004

[vbab041-B10] Gregori J. et al (2021) *msmsEDA: Exploratory Data Analysis of LC-MS/MS Data by Spectral Counts*. R package version 1.30.0.

[vbab041-B11] Hastings J. et al (2016) ChEBI in 2016: improved services and an expanding collection of metabolites. Nucleic Acids Res., 44, D1214–D1219. 26467479 10.1093/nar/gkv1031 PMC4702775

[vbab041-B12] Kanehisa M. , Goto S. (2000) KEGG: kyoto encyclopedia of genes and genomes. Nucleic Acids Res., 28, 27–30. 10592173 10.1093/nar/28.1.27 PMC102409

[vbab041-B13] Karpievitch Y. et al (2009) A statistical framework for protein quantitation in bottom-up MS-based proteomics. Bioinformatics, 25, 2028–2034. 19535538 10.1093/bioinformatics/btp362 PMC2723007

[vbab041-B14] Kuzniar A. , Kanaar R. (2014) PIQMIe: a web server for semi-quantitative proteomics data management and analysis. Nucleic Acids Res., 42, W100–W106. 24861615 10.1093/nar/gku478 PMC4086067

[vbab041-B15] Minadakis G. et al (2020) ProtExA: a tool for post-processing proteomics data providing differential expression metrics, co-expression networks and functional analytics. Comput. Struct. Biotechnol. J., 18, 1695–1703. 32670509 10.1016/j.csbj.2020.06.036 PMC7340977

[vbab041-B16] Pham T.V. et al (2020) iq: an R package to estimate relative protein abundances from ion quantification in DIA-MS-based proteomics. Bioinformatics, 36, 2611–2613. 31909781 10.1093/bioinformatics/btz961 PMC7178409

[vbab041-B17] Piovesan D. et al (2021) MobiDB: intrinsically disordered proteins in 2021. Nucleic Acids Res., 49, D361–D367. 33237329 10.1093/nar/gkaa1058 PMC7779018

[vbab041-B18] Puchala W. et al (2020) HaDeX: an R package and web-server for analysis of data from hydrogen-deuterium exchange mass spectrometry experiments. Bioinformatics, 36, 4516–4518. 32579220 10.1093/bioinformatics/btaa587 PMC7575049

[vbab041-B19] Ritchie M.E. et al (2015) limma powers differential expression analyses for RNA-sequencing and microarray studies. Nucleic Acids Res., 43, e47. 25605792 10.1093/nar/gkv007 PMC4402510

[vbab041-B20] Ritz C. et al (2015) Dose-response analysis using R. PLoS One, 10, e0146021. 26717316 10.1371/journal.pone.0146021 PMC4696819

[vbab041-B21] Shah A.D. et al (2020) LFQ-analyst: an easy-to-use interactive web platform to analyze and visualize label-free proteomics data preprocessed with MaxQuant. J. Proteome Res., 19, 204–211. 31657565 10.1021/acs.jproteome.9b00496

[vbab041-B22] Szklarczyk D. et al (2019) STRING v11: protein-protein association networks with increased coverage, supporting functional discovery in genome-wide experimental datasets. Nucleic Acids Res., 47, D607–D613. 30476243 10.1093/nar/gky1131 PMC6323986

[vbab041-B23] The UniProt Consortium (2021) UniProt: the universal protein knowledgebase in 2021. Nucleic Acids Res., 49, D480–D489. 33237286 10.1093/nar/gkaa1100 PMC7778908

[vbab041-B24] Tyanova S. , Cox J. (2018) Perseus: a bioinformatics platform for integrative analysis of proteomics data in cancer research. Methods Mol. Biol., 1711, 133–148. 29344888 10.1007/978-1-4939-7493-1_7

[vbab041-B25] van Breukelen B. et al (2009) StatQuant: a post-quantification analysis toolbox for improving quantitative mass spectrometry. Bioinformatics, 25, 1472–1473. 19336442 10.1093/bioinformatics/btp181

[vbab041-B26] Wickham H. et al (2019) Welcome to the tidyverse. J. Open Source Softw., 4, 1686.

[vbab041-B27] Wieczorek S. et al (2017) DAPAR & ProStaR: software to perform statistical analyses in quantitative discovery proteomics. Bioinformatics, 33, 135–136. 27605098 10.1093/bioinformatics/btw580 PMC5408771

[vbab041-B28] Willforss J. et al (2019) NormalyzerDE: online tool for improved normalization of omics expression data and high-sensitivity differential expression analysis. J. Proteome Res., 18, 732–740. 30277078 10.1021/acs.jproteome.8b00523

[vbab041-B29] Zhang X. et al (2018) Proteome-wide identification of ubiquitin interactions using UbIA-MS. Nat. Protoc., 13, 530–550. 29446774 10.1038/nprot.2017.147

